# Bilateral multifocal chorioretinitis as the only presentation of acute West Nile virus infection: a case report

**DOI:** 10.1186/s12886-024-03423-8

**Published:** 2024-04-10

**Authors:** Nicola Valsecchi, Chiara Veronese, Matilde Roda, Antonio Pasquale Ciardella, Luigi Fontana

**Affiliations:** 1 Ophthalmology Unit, Dipartimento Di Scienze Mediche E Chirurgiche, Alma Mater Studiorum University of Bologna, Bologna, Italy; 2 IRCCS Azienda Ospedaliero-Universitaria Di Bologna, Bologna, Italy

**Keywords:** West Nile virus, Chorioretinitis, Acute infection, Uveitis, Case report

## Abstract

**Background:**

To describe a case of bilateral multifocal chorioretinitis as the only presentation of acute West Nile virus (WNV) infection in the absence of neurological involvement.

**Case presentation:**

A 78-year-old Italian woman was admitted to our emergency department because she noticed blurry vision in both eyes. She did not report fever, fatigue, or neurological symptoms in the last few days. Multimodal imaging showed the presence of bilateral hyperfluorescent lesions with a linear distribution, that corresponded to hypocyanescent spots on indocyanine green angiography. Antibody serology showed the presence of IgM antibodies, IgG antibodies, and ribonucleic acid (RNA) for WNV. Magnetic resonance imaging (MRI) of the brain ruled out central nervous system involvement. Three months later, the patient reported spontaneous resolution of her symptoms and remission of the chorioretinal infiltrates.

**Conclusions:**

In endemic areas, it is important to think of acute WNV infection as an explanatory etiology in cases of multifocal chorioretinitis, even without neurological involvement.

## Background

West Nile virus (WNV) is a single-stranded ribonucleic acid (RNA) flavivirus transmitted mainly by the bite of infected mosquito species of the genus *Culex* and *Aedes spp *[1].

Human infections show a seasonal trend, with most cases reported between July and October, with peaks in late August [2]. Nearly 80% of WNV infections in humans are asymptomatic, up to 20% of infections present with a mild flu-like syndrome, and less than 1% of WNV infections develop a neuroinvasive disorder. When the central nervous system is involved, the mortality rate is about 10% [3].

Multifocal chorioretinitis is a common ocular manifestation of WNV infection with neuroinvasive disease, which is frequently asymptomatic and self-limiting [4] However, involvement of the choroid and retina is rare in patients with asymptomatic systemic disease [5–7].

The present study aims to describe a case of bilateral multifocal chorioretinitis as the only presentation of acute WNV infection.

### Case presentation

**Fig. 1:**
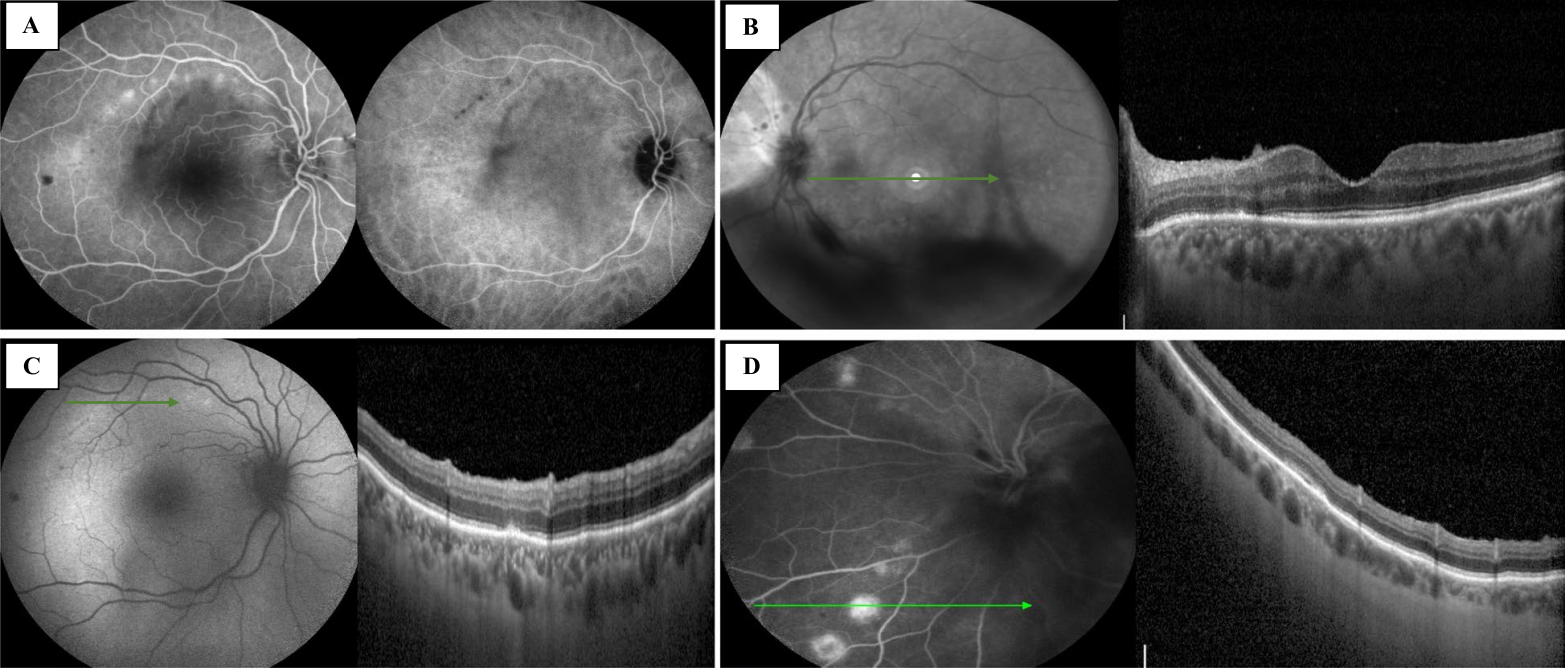
Multimodal imaging. **A** In the right eye, fluorescein angiography (FA) showed the presence of hyperfluorescent lesions with a linear distribution, corresponding to the hypocyanescent lesions in indocyanine green angiography (ICGA). **B** In the left eye, FAF showed the presence of vitreitis, while OCT showed the presence of hyperreflective dots in the vitreous chamber, hyperreflective oval deposits on the retinal surface, focal alteration in the retinal pigmented epithelium (RPE) and the ellipsoid zone (EZ), and granular hyperreflective specks located predominantly in the outer and inner nuclear layers.** C** In the right eye, fundus autofluorescence (FAF) showed the presence of hyperautofluorescent lesions with a linear distribution, corresponding to focal alteration in the retinal pigmented epithelium (RPE) and the ellipsoid zone (EZ) at the OCT. **D** In the left eye, FA showed the presence of “target” lesions with a rim of hyperfluorescence and a central spot of hypofluorescence, corresponding to focal alteration of the RPE and the EZ

**Fig. 2:**
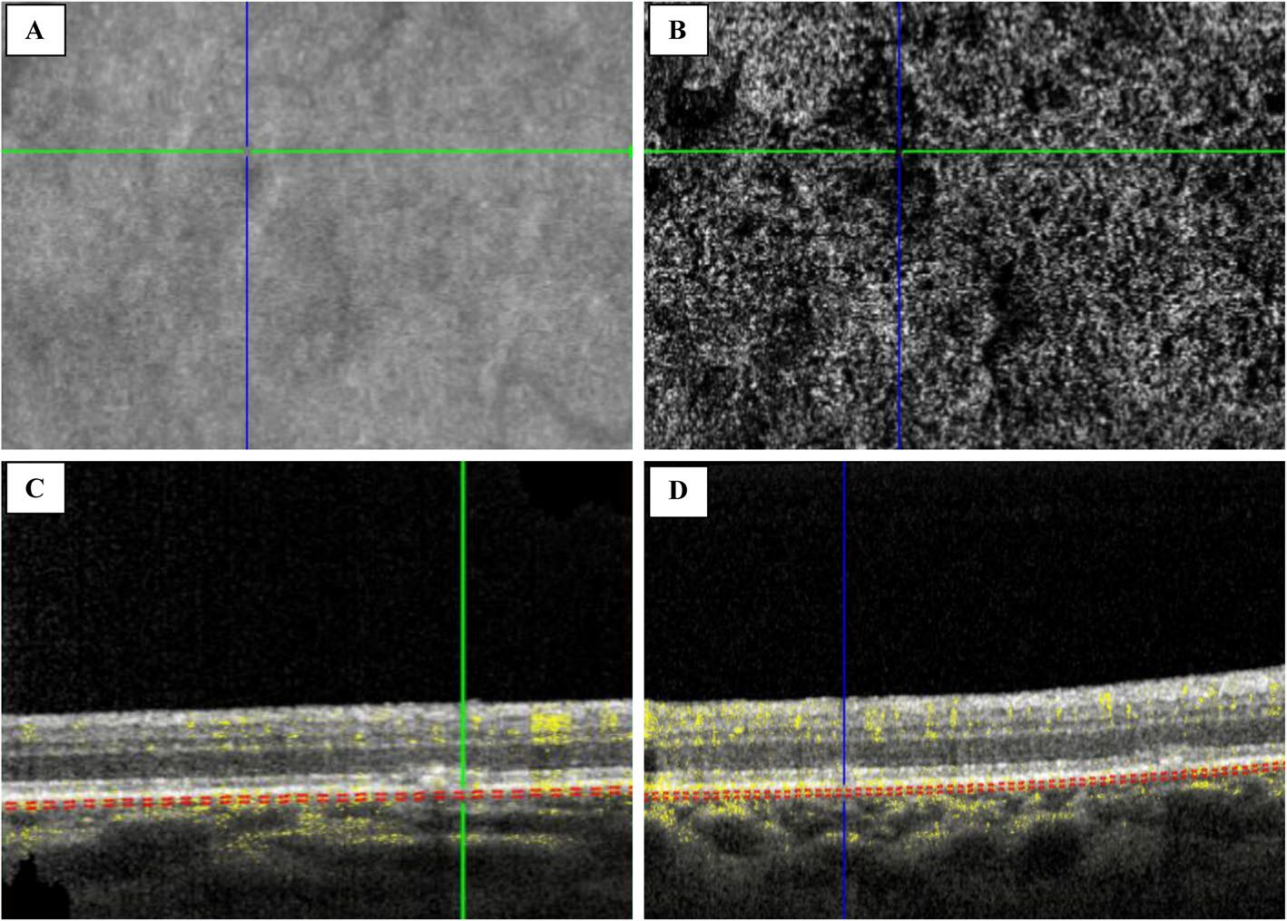
Optical Coherence Tomography Angiography (OCTA) at the level of the lesions in the right eye. **A** OCT en face. **B** OCTA showed diffuse capillary network attenuation in the choriocapillaris at the level of the lesions. **C** and **D** OCT at the level of the lesions

A 78-year-old Caucasian woman was admitted to the ophthalmic emergency room of Sant’Orsola-Malpighi Hospital (Bologna, Italy) because she noticed blurry vision in both eyes. The patient lived in a small rural town in the district of Bologna in the Emilia Romagna region. She was not under medical treatment. She did not have diabetes mellitus and she was immunocompetent. In addition, her vital signs were normal, and she did not report fever, fatigue, or neurological symptoms in the last few days, except for the onset of blurred vision in both eyes, which started in the last week. Best-corrected visual acuity (BCVA) was 20/30 in both eyes, there were no relative afferent pupillary defects (RAPD), intraocular pressure was normal, and there was mild flare in the anterior chamber in both eyes. Fundoscopy examination revealed bilateral vitreitis (grade 1 + , according to NIH grading system [8]) and bilateral swelling of the optic disc. Computed Tomography (CT) scan of the brain was immediately performed, revealing no pathological findings. In accordance with the clinical scenario, a standard workup for uveitis was immediately requested. Waiting for the laboratory test results, empirical therapy with topical dexamethasone in both eyes twice daily was started. During the next few days, the diagnostic workup was completed. Visual field test was performed and revealed a mild physiological blind spot enlargement on both eyes; spectral-domain optical coherence tomography (SD-OCT) (Spectralis OCT, Heidelberg Engineering, Germany) showed the presence of oval hyperreflective deposits on the retinal surface in the left eye, focal alterations in the retinal pigment epithelium (RPE) and the ellipsoid zone (EZ), and granular hyperreflective specks located predominantly within the outer and inner nuclear layers in both eyes (Figs. 1A and B). Fluorescein angiography (FA) showed the presence of hyperfluorescent lesions in the late phase with a linear distribution, corresponding to the hypocyanescent spots on indocyanine angiography (ICGA) (Figs. 1C and D). FA was more sensitive in detecting lesions than ICGA. In both eyes, the OCT scan at the level of the lesion showed the presence of alterations in the RPE and EZ (Figs. 2A and B). Optical Coherence Tomography Angiography (OCTA) showed diffuse attenuation of the capillary network in the choriocapillaris at the level of the lesions, with no evidence of alterations at the level of the superficial and deep capillary plexus (Figs. 2C and D). Considering the characteristic linear pattern of the chorioretinal lesions, antibody serology and serum RNA for West Nile virus, Toscana virus, and Usutu virus were also requested.

Antibody results were available the following week. The Quantiferon-TB Gold test, Venereal Disease Research Laboratory, and Treponema pallidum particle agglutination test were negative. In addition, IgM antibodies were negative for Herpes simplex virus type-1, Herpes simplex virus type-2, Epstein-Barr virus, Cytomegalovirus, Varicella-zoster virus, and Toxoplasma. However, the antibody serology detected IgM antibodies, IgG antibodies, and RNA for West Nile virus.

Therefore, the patient was immediately contacted and referred to the infectious diseases department, where she underwent magnetic resonance imaging (MRI) of the brain, which ruled out central nervous system involvement.

Hence, asymptomatic West Nile Virus infection with bilateral multifocal chorioretinitis was diagnosed.

**Fig. 3:**
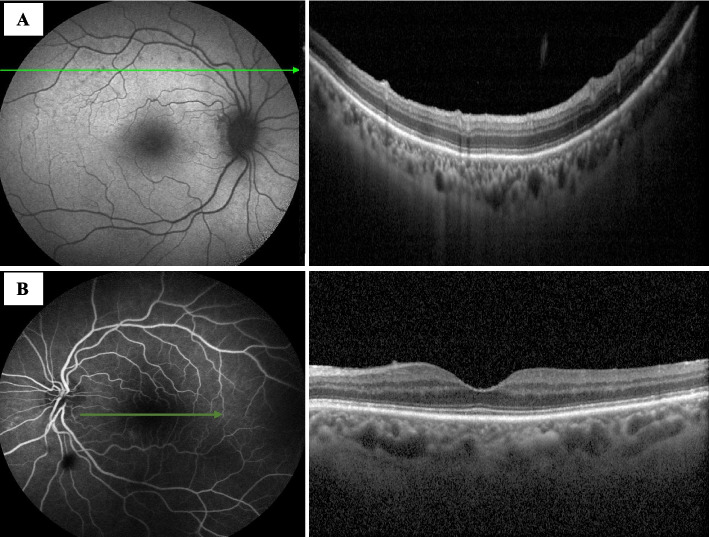
One-month follow-up. **A** In the right eye, OCT at the level of the lesions showed recovery of the RPE and the EZ. **B** In the left eye, OCT showed a reduction in the hyperreflective oval deposit on the retinal surface in the left eye

One month after the first ophthalmological examination, visual acuity in both eyes was 20/25, there was no involvement of the anterior segment and vitreitis was absent. OCT showed a reduction of the oval hyperreflective deposits on the retinal surface in the left eye. In addition, FA and ICGA scans in both eyes showed reduced hyperfluorescent spots in the late phase (Figs. 3A and B). OCT showed recovery of the EZ and RPE at the level of the lesions (Fig. 3C and D), and OCTA showed a resolution of the attenuation of the capillary network in the choriocapillaris at the level of the lesions. In accordance with the clinical scenario, topical dexamethasone was discontinued. No adverse effects occurred.

Three months after the initial evaluation, the patient reported an improvement in her visual acuity. Visual acuity was stable at 20/25 in both eyes, and there was no evidence of active chorioretinitis on fundoscopic examination.

## Discussion and conclusions

WNV infection is an emerging disease worldwide and an endemic infection in Italy. In 2022, 586 cases of WNV infection were registered in Italy, representing 60,7% of the total human WNV infections in Europe [9]. Emilia Romagna and Veneto, in north-eastern Italy, are the regions most affected by WNV infections. This condition depends mainly on native mosquitoes, the presence of susceptible endemic birds, and local environmental conditions [2]. Moreover, human cases of WNV infection have consistently risen in Italy and Europe [10].

In the present study, we describe the case of a patient who presented with bilateral multifocal chorioretinitis as the only presentation of acute West Nile virus infection, which required infectious disease consultation to rule out central nervous system involvement. Multifocal chorioretinitis is rare in patients with asymptomatic systemic infection. Chorioretinal involvement is mainly related to neuroinvasive cases, which are associated with high mortality, and a high risk of neurological complications. Hasbun et al. 2016 reported only one case of retinal involvement in 26 cases of asymptomatic WNV infection [6]. In addition, Sanz et al. 2020 showed one case of unilateral chorioretinitis secondary to WNV infection in an asymptomatic patient with an eighteen-month history of photopsia and visual disturbances in her left eye [7]. Multifocal chorioretinitis is the most frequent ocular manifestation of WNV infection. However, several ophthalmologic findings have been reported, including iridocyclitis, retinal vasculitis, macular edema, choroidal neovascularization, optic neuropathy, neuroretinitis, congenital chorioretinal scarring, ocular nerve palsy, and nystagmus secondary to encephalitis [5, 11, 12]. Excluding the most severe cases, West Nile chorioretinitis is considered to be a self-limiting pathology if retinal and choroidal complications do not occur, and patients can be watched without treatment. Topical steroids and mydriatic agents can be used for anterior uveitis, while peripheral retinal photocoagulation of the ischemic areas of the retina can effectively prevent neovascularization. In addition, anti-vascular endothelial growth factor agents can be used to treat choroidal neovascularization, whereas surgical treatment can be required for vitreous hemorrhage or tractional retinal detachment [12]. In cases of systemic involvement, there is no effective treatment for WNV infection, and supportive care remains the mainstay of treatment. The use of systemic steroids in WNV infections is controversial, and their utilization is reserved only in specific cases of potentially life-threatening neuroinvasive forms [13]. Systemic steroid use carries the risk of elevating an immunosuppressive state, potentially exacerbating WNV viremia and worsening patient outcomes. Moreover, prior studies have not demonstrated a clear advantage of steroid treatment in cases of WNV encephalitis [14]. However, recent experimental and clinical evidence suggests that pro-inflammatory mediators might play a role in WNV pathogenesis, and some case series have documented the administration of high-dose steroids leading to improved outcomes in patients with WNV neuroinvasive disease [15]. Nonetheless, no randomized clinical trials have been conducted to assess the efficacy of systemic steroids in managing acute life-threatening infections and neurological sequelae in WNV infections, and there is no scientific evidence that support the role of systemic steroids in treating posterior uveitis, as WNV chorioretinitis typically follows a self-limiting course, with most patients regaining their baseline visual acuity***.*** Other endemic viruses can cause chorioretinal involvement. Post-fever retinitis is an infectious or para-infectious uveitic entity caused by several viral, or bacterial agents commonly seen in tropical countries. Ocular manifestations typically start developing within days to weeks after the onset of febrile illness, with different clinical features [16, 17]. For instance, Rift Valley Fever virus can determine bilateral retinitis and occlusive vasculitis in the posterior pole, preceded by a fever in an endemic area [18]. In the present case, the patient presented with pathognomonic linear lesions extending towards the periphery, following the retinal nerve fiber layer, suggestive of WNV infection. OCTA showed no evidence of superficial and deep retinal capillary ischemia, but attenuation of the choriocapillaris network at the lesion level. In 2017, Khairallah et al. described capillary rarefaction in the superficial and deep capillary plexus in a patient with occlusive retinal vasculitis associated with WNV infection [19]. Our findings support the hypothesis of a primary location of disease activity at the level of the outer retina and RPE, with hematogenous spread of the virus via the choriocapillaris. We also observed granular hyperreflective specks located predominantly in the outer and inner nuclear layers, as previously described [20].

Another interesting finding in the present case was the presence of a single hyperreflective oval deposit on the retinal surface in the patient's left eye, which diminished in size after the active infection subsided. Hyperreflective oval deposits located on the retinal surface have been previously described in toxoplasmic posterior uveitis and viral retinitis. This case would be the first to describe the presence of hyperreflective oval deposits in West Nile virus infection [21].

To conclude, in the present case, we described the presence of bilateral multifocal chorioretinitis as the only presentation of acute WNV infection. Therefore, in endemic areas, it is crucial to consider WNV infection as an explanatory etiology in cases of multifocal chorioretinitis, even without neurological involvement.

## Data Availability

The data that support the findings of this study are available on request from the corresponding author.
